# Impaired neuronal activity as a potential factor contributing to the underdeveloped cerebrovasculature in a young Parkinson’s disease mouse model

**DOI:** 10.1038/s41598-023-49900-w

**Published:** 2023-12-18

**Authors:** Jin-Young Jeong, Hyun Jung Lee, Namsuk Kim, Yan Li, Jong-Cheol Rah, Won-Jong Oh

**Affiliations:** 1 Neurovascular Biology Laboratory, Neurovascular Unit Research Group, Korea Brain Research Institute, Daegu, 41062 South Korea; 2 Sensory and Motor System Research Group, Korea Brain Research Institute, Daegu, 41062 South Korea; 3 Department of Brain Sciences, Daegu Gyeongbuk Institute of Science and Technology, Daegu, 42988 South Korea

**Keywords:** Neuroscience, Diseases

## Abstract

Misfolding of α-synuclein (α-Syn) in the brain causes cellular dysfunction, leading to cell death in a group of neurons, and consequently causes the progression of Parkinson’s disease (PD). Although many studies have demonstrated the pathological connections between vascular dysfunction and neurodegenerative diseases, it remains unclear how neuronal accumulation of α-Syn affects the structural and functional aspects of the cerebrovasculature to accelerate early disease progression. Here, we demonstrated the effect of aberrant α-Syn expression on the brain vasculature using a PD mouse model expressing a familial mutant form of human α-Syn selectively in neuronal cells. We showed that young PD mice have an underdeveloped cerebrovasculature without significant α-Syn accumulation in the vasculature. During the early phase of PD, toxic α-Syn was selectively increased in neuronal cells, while endothelial cell proliferation was decreased in the absence of vascular cell death or neuroinflammation. Instead, we observed altered neuronal activation and minor changes in the activity-dependent gene expression in brain endothelial cells (ECs) in young PD mice. These findings demonstrated that neuronal expression of mutant α-Syn in the early stage of PD induces abnormal neuronal activity and contributes to vascular patterning defects, which could be associated with a reduced angiogenic potential of ECs.

## Introduction

It has been widely recognized that impairments in vascular structure and function are critical to the pathogenesis of many neurodegenerative diseases, including Parkinson’s disease (PD)^1,2^. Studies in PD patients and animal models have shown diverse degrees of cerebrovascular damage, such as a high risk of ischemic stroke, incidence of cerebral microbleeds, and blood‒brain barrier (BBB) dysfunction^3–6^. To date, two distinct histological findings have been reported regarding cerebrovascular formation in PD. First, pathological angiogenesis leads to increased vascular density that is frequently observed in postmortem PD brains and various animal models, and is likely to occur in the moderate stage of PD^7^. Consistent with these findings, the upregulation of various angiogenic markers, such as vascular endothelial growth factor, has been reported in PD patients and nonhuman primates^8,9^. However, other findings have shown that vascular degeneration occurs in patients with PD or Lewy body dementia and, in some cases, is accompanied by an increased occurrence of string vessels, a thin connective tube devoid of endothelial cells (ECs)^10,11^. It appears that the devastating loss of ECs resulting in decreased cerebrovascular density occurs at a comparatively late stage in PD pathology.

PD is typically characterized by neuronal damage; in particular, a selective loss of dopaminergic neurons of the substantia nigra pars compacta due to abnormal accumulation of α-synuclein (α-Syn) protein, hence representing a synucleinopathy^12^. α-Syn is a 14-kilodalton (kDa) protein that normally acts as a neuronal presynaptic component in the regulation of neurotransmitter release, synaptic function and neuroplasticity^13^. Under various pathological conditions, α-Syn changes its structure from monomeric to a higher-order structure, such as fibrillar or oligomeric forms, and can be transported intercellularly in the brain as well as from the periphery^12,14,15^. Genetically, multiplications and various point mutations of the α-Syn gene lead to dominant familial parkinsonism. These symptoms are rapidly triggered by abnormal aggregation processes^12,16^. Rodent models of PD have been developed based on genetic mutations, many of which show cerebrovascular defects including hypoperfusion and BBB disruption before the onset of severe neuronal and motor symptoms^6^. However, the mechanisms underlying these vascular changes remain unclear.

Here, we investigated vascular network formation in the M83 PD mouse model that selectively overexpresses a familial mutant form of human α-Syn (A53T) in neuronal cells. We observed that the early stages of the PD model show underdeveloped vascular patterning that was not caused directly by toxic α-Syn in vessels, but rather by indirect inhibition of angiogenic processes mediated by neuronal activation. Our findings provide new insights into how aberrant neuronal activation due to α-Syn toxicity in early PD can affect the brain vasculature.

## Materials and methods

### Mouse models

M83 transgenic mice (JAX stock #004479)^17^ and SNCA KO mice (JAX stock #016123) were purchased from the Jackson Laboratory and maintained on a C57Bl/6 J (JAX stock #000664) background. Specifically, M83 mice were backcrossed to C57Bl/6 J mice for more than 10 generations to generate a congenic strain. Mice were housed under standard conditions with a 12-h light/dark cycle and free access to food and water. All protocols for animal experiments were approved by the Institutional Animal Care and Use Committee of the Korea Brain Research Institute (IACUC-19–00001 and IACUC-20–00011). All experiments were performed under the National Institutes of Health Guide for the Care and Use of Laboratory Animals and ARRIVE guidelines.

### Blood vessel labeling and tissue clearing

To label cerebral blood vessels, we injected 100 μg of fluorescence-labeled tomato lectin (cat# DL-1174, Vector Laboratories) into the retro-orbital sinus of the mouse. After 5 min of circulation, the mice were subjected to cardiac perfusion and postfixation with 4% paraformaldehyde in phosphate-buffered saline (4% PFA/0.1 M PBS, pH 7.4) at 4 °C for 2 h, and then the tissue was cleared according to the previous protocol^18,19^. Briefly, brain samples sliced at 3 mm thickness were dehydrated with ethanol (EtOH) (30%, 50% and 70% EtOH, pH 9.0 with 2% Tween 20, followed by 100% EtOH). For tissue dehydration, tissues were incubated with 100% EtOH twice every 24 h at 4 °C. The samples were then transferred to ethyl cinnamate (ECi; cat# 112372, Sigma-Aldrich) and incubated at room temperature (RT) with gentle shaking until they became transparent, and cleared samples were stored in ECi at RT until imaging. All procedures were performed under dark conditions.

### Immunohistochemistry (IHC)

For immunostaining with floating samples, brains were fixed overnight in 4% PFA in phosphate buffer (4% PFA/0.1 M PB, pH 7.4) and equilibrated with 30% sucrose in 1 × PB. After equilibration, brains were embedded in O.C.T. compound (cat# 3801480, Leica BIOSYSTEMS). Mouse brain slices were cut into 30 μm slices on a cryostat (Leica Microsystems Inc.). Brain slices were washed in PBS to remove residual O.C.T. compound and permeabilized in PBS-T (PBS containing 0.2% Triton X-100) for 5 min. They were then blocked with 1% bovine serum albumin (BSA) in PBS-T (1% BSA/PBS-T) containing 0.3 M glycine for 1 h at RT and incubated with primary antibodies diluted in 1% BSA/PBS-T at 4 °C overnight. The following primary antibodies were used at the indicated dilutions: anti-α-synuclein (MJFR1, 1:10,000, cat# ab138501, Abcam), anti-α-synuclein (Syn211, 1:1,000, cat# 36-008, Merck), anti-α-synuclein phospho-Ser129 (1:20,000, cat# 825701, BioLegend), anti-CD31 (1:500, cat# 553370, BD Bioscience), anti-NeuN (1:20,000, cat# ab177487, Abcam), anti-cleaved caspase-3 (1:1,000, cat# 9661, Cell Signaling), anti-BrdU (1:500, cat# ab6326, Abcam), anti-ERG (1:100, cat# ab92513, Abcam), anti-c-fos (1:2,000, cat# ab190289, Abcam), anti-vGluT2 (1:5,000, cat# AB2251-I, Millipore), anti-GFAP (1:500, cat# Z0334, Dako) and anti-Iba1 (1:500, cat# ab178847, Abcam). After three washes with PBS-T, sections were incubated with Alexa Fluor 488-, 594-, or 647-conjugated secondary antibodies (1:1,000, Invitrogen) for 1 h under light protection conditions. Unbound secondary antibodies were removed with three washes with PBS-T. Stained brain sections were mounted with ProLong™ Diamond Antifade Mountant with DAPI (cat# P36962, Invitrogen) for imaging analysis. To reduce nonspecific autofluorescence in the aged tissue samples, 1 × Trueblack solutions (cat# 23007, Biotium) diluted in 70% ethanol were treated for 30 s, followed by thorough washing in 1 × PBS before mounting^20^. For whisker stimulation, P10 pups were gently held by their bodies and their right whiskers were manually stimulated for 15 min (3–4 Hz) using a paintbrush, after which the fixation process was performed as described in the above Sect. ^21^.

### Tissue lysate preparation and Western blot

The tissue preparation procedure was adapted from a previous study^22^. Briefly, the brain samples were homogenized in lysis buffer [50 mM Tris–HCl (pH 7.4), 150 mM NaCl, 1 mM ethylenediamine tetraacetic acid (EDTA), 1% Triton X-100, 0.5% sodium dodecyl sulfate (SDS), 0.5% sodium deoxycholate] supplemented with protease inhibitor cocktail (cat# 05892970001, Roche) and phosphatase inhibitor cocktail (cat# 04906837001, Roche) using a Taco™Prep Bead Beater (GeneReach, Biotechnology Corp., Taiwan). After homogenization, the samples were rotated at 4 °C for 30 min for complete lysis. The homogenate was then centrifuged at 15,000 × g for 20 min, and the protein contents of the supernatants were quantified using a Pierce™ BCA Protein Assay Kit (cat# 23225, Thermo Fisher Scientific). Samples were separated on SDS–polyacrylamide gels and transferred to nitrocellulose or polyvinylidene fluoride (PVDF) membranes. The membranes were blocked with 5% skim milk in Tris-buffered saline containing 0.1% Tween-20 (TBS-T) for 1 h. Specifically, for the detection of tight junction proteins, Everyblot blocking buffer (cat# 12010020, Bio-Rad) was used, and the membranes were incubated with the indicated primary antibodies described below in the same blocking buffer at 4 °C overnight. The primary antibodies were anti-α-synuclein (1:3,000, cat# sc12767, Santa Cruz Biotech), anti-α-synuclein (phospho S129) (1:3,000, cat# ab168381, Abcam), anti-ZO-1 (1:1,000, cat# 61-7300, Invitrogen), anti-claudin-5 (1:1,000, cat# 34-1600, Invitrogen), anti-occludin (1:1,000, cat# 71-1500, Invitrogen), anti-CD31 (1:1,000, cat# AF3628, R&D Systems), and anti-β-actin (1:5,000, cat# 5125, Cell Signaling). After incubation with appropriate HRP-conjugated secondary antibodies, protein bands were visualized by enhanced chemiluminescence using Fusion FX7 (Vilber, Germany) and analyzed using ImageJ. To detect phospho-α-Syn expression, an additional membrane fixation step was added^23^. Before blocking, the membrane was fixed with 4% PFA (PBS)/0.01% glutaraldehyde for 30 min. Then, the PFA was washed with TBST three times every 10 min. Raw data from Western blot images are included in a separate supplementary file.

### Dot blot for protein aggregation assay

Brain tissue was homogenized with extraction buffer [10 mM Tris–HCl (pH 7.4), 0.5 mM dithiothreitol (DTT), 5 mM MgCl_2_, and 3.08 mg/ml adenosine triphosphate (ATP)] supplemented with a protease inhibitor cocktail (cat# 05892970001, Roche) using a Taco™ Prep Bead Beater (GeneReach, Biotechnology Corp., Taiwan). After homogenization, tissue homogenates were placed on ice for 30 min to lyse the tissue. Homogenates were transferred to new microcentrifuge tubes and centrifuged at 15,000 × g for 90 min at 4 °C. The supernatant (soluble proteins) was moved into a new microcentrifuge tube and quantified by BCA assay. To denature protein aggregates, a solution of 50% formic acid (cat# 695076, Sigma-Aldrich) was mixed with tissue homogenates and incubated for 1 h at 37 °C following the manufacturer’s instructions for the anti-α-synuclein aggregate antibody (cat# ab209538, Abcam). Overall dot blotting and analysis procedures followed a previous protocol^24^. Briefly, before applying the sample, the nitrocellulose (NC) membrane was dampened in Tris-buffered saline (TBS) for a minimum of 10 min, and the apparatus was assembled. The detailed assembly order followed the manufacturer's instructions (Bio-Dot Microfiltration Apparatus, cat# 170-6545, Bio-Rad). After rehydrating the membrane with 100 μL of TBS, samples were added to each well of the membrane in triplicate, and the apparatus was covered with foil to prevent contamination. The apparatus was then allowed to sit for at least 1 h without vacuum, and any remaining samples were eliminated by vacuum. The membrane was removed from the apparatus and dried for 1 h. The membrane was incubated with a blocking solution (5% skim milk with 0.1% TBS-T) for 1 h at RT. After discarding the blocking solution, the primary antibody dissolved in the blocking solution was added to the membrane and incubated at 4 °C overnight. The membrane was probed with the appropriate horseradish peroxidase (HRP)-conjugated secondary antibody and detected by SuperSignal™ West Pico PLUS Chemiluminescent Substrate (cat# 34580, Thermo Scientific). To detect whole proteins as a control, the dried membrane was immersed in Ponceau S solution (cat# P7170, Sigma-Aldrich) before blocking for 5 min, followed by three rinses in distilled water. The following antibodies were used: anti-α-synuclein (1:3,000, cat# 610787, BD Biosciences) and anti-α-synuclein aggregate antibody (2.2 ng/ml, cat# ab209538, Abcam). Raw data from dot blot images are included in a separate supplementary file.

### Cell proliferation analysis

100 μg/g bromodeoxyuridine (BrdU) (cat #ab142567, Abcam) was injected one day before sacrifice. Since BrdU is incorporated into the nucleus of a proliferating cell, the DNA hydrolysis step must be performed first. Briefly, fixed tissue sections were washed three times every 5 min with 1% Triton X-100/PBS. Then, 1 N HCl solution was applied to the tissues for 10 min on ice to lyse the DNA structure. This was followed by 2 N HCl for 10 min at RT, and then tissues were transferred to an incubator at 37 °C for 20 min. Immediately after removal of HCl, 0.1 M borate buffer (100 mM boric acid and 25 mM NaOH, pH 8.5) was added for neutralization for 12 min at RT. The samples were then washed three times every 5 min in 1% Triton X-100/PBS, and further immunostaining was performed as described above.

### Imaging and analyses

A light-sheet microscope with a 5 × EC plan objective lens and LightsheetZ.1 software (Carl Zeiss, Germany) were used to image whole brains. Imaris 9.0.2 (Bitplane) was used for image reconstruction. Three-dimensional rendering of light-sheet image data and quantification of cerebral blood vessels (length and branch points) were performed using Imaris software. Images of brain slices were acquired using a confocal microscope (A1 Rsi/Ti-E, Nikon). All image processing was performed using Fiji (ImageJ) and Adobe Photoshop (Adobe Photoshop CC 2022) software.

### Capillary isolation and RNA purification

The Mouse somatosensory cortex was isolated in chilled Dulbecco's Modified Eagle Medium/1% penicillin‒streptomycin (DMEM/1% P/S), and capillary isolation was performed to prepare RNA in accordance with a previously established protocol^25^. Briefly, tissues were dissociated by using a glass homogenizer (cat# 357542, WHEATON) in 10 mL of chilled DMEM/1% P/S and centrifuged at 1000 × g for 10 min at 4 °C. After discarding the supernatants, the pellets were resuspended in 15 mL of 20% BSA/DMEM/1% P/S to avoid myelin contamination and centrifuged at 2,500 × g for 10 min at 4 °C. This step was repeated one time. After discarding the supernatants, the pellets were resuspended in 5 mL of chilled PBS and passed through a 40-μm cell strainer (cat# 93040, SPL Life Sciences). The strainer mesh containing the vessels was then cut with a disposable scalpel (cat# 371611, Bard-Parker) and transferred to a microcentrifuge tube. One mL of TRIzol reagent (cat# 15596026, Invitrogen) was added to the mesh, followed by 200 µL of chloroform (cat# C2432, Sigma-Aldrich). After centrifugation at 12,000 × g for 15 min at 4 °C, the upper aqueous band was transferred to a new tube and mixed with RNA precipitation solution [60 μL 4 M LiCl (cat# 213,233, Sigma-Aldrich), 120 μL 20 × TE (cat# A2651, Promega), 1.8 mL 100% EtOH (cat# E7023, Sigma-Aldrich), 3 μL glycogen (cat# 10901393001, Roche)]. Samples were incubated at -20 °C overnight. The next day, the samples were centrifuged at 12,000 × g for 10 min at 4 °C, and the RNA pellets were washed with 1 mL 75% EtOH before air drying at RT. The dried pellets were then resuspended in 16 µL of RNase-free water. To remove the genomic DNA from the sample, DNase I (cat# 18068-015, Invitrogen) was added according to the manufacturer's instructions. To obtain highly purified samples, another round of RNA precipitation was performed using the procedures described above, except that the reagent volume was reduced. The amount of isolated mRNA was measured using the Agilent 4200 TapeStation System (Agilent Technologies) according to the manufacturer's instructions.

### cDNA synthesis and quantitative real-time PCR (RT‒qPCR)

Total RNA was used for cDNA synthesis with the Transcriptor First Strand cDNA synthesis kit (cat# 4896866001, Roche) according to the manufacturer’s instructions. RT–qPCR was performed using LightCycler 480 SYBR Green I Master Mix (cat# 04707516001, Roche) on a LightCycler 480 II system (Roche). Reactions were run in technical triplicates, of which the mean was determined before subsequent analysis. The relative expression of each gene was calculated using the 2^−ΔΔCt^ method and normalized to *Gapdh*. The primers used in this study were designed using Primer3 software, and their sequences are shown in Supplementary Table S1.

### BBB permeability test

To assess BBB permeability, 100 μg of 1 kDa cadaverine-594 (cat# A30678, Invitrogen) was injected retro-orbitally. After a 2 h circulation period, the mice were anesthetized with avertin (125 mg/kg). Blood and brain were collected separately as described below. After puncture of the right atrium, approximately 300 μL blood was collected, kept on ice for 15 min, and centrifuged at 10,000 × g for 10 min at 4 °C. The serum portion was mixed with 20% trichloroacetic acid (TCA) at a 1:2 ratio (serum:TCA) and centrifuged at 12,000 × g for 20 min at 4 °C. For the brain tissue, the heart was perfused with PBS, and then the brain was extracted. After the brains were weighed, 20% TCA was added to the tissue at a 1:2 ratio (brain:TCA). The homogenates were diluted with an equal volume of 1% Triton X-100/PBS and centrifuged at 12,000 × g for 20 min at 4 °C. To measure the fluorescence intensity in both the brain and serum supernatant, 30 μL supernatant was mixed with 90 μL 95% EtOH and loaded into a 96-well black plate (cat# 33396, SPL Life Sciences). Fluorescence intensity was measured using a FlexStation 3 (Molecular Device)^26^. The degree of BBB breakdown was described as the permeability index^27^. To analyze BBB permeability at the histological level, a lysine-fixable dye, Texas red dextran (MW = 10 kDa, Invitrogen; 0.1 ml 10 mg/ml; cat# D1863, Invitrogen), was injected retro-orbitally. After 10 min of dye circulation, the brains were removed and fixed in 4% PFA overnight, followed by equilibration with 30% sucrose in 1 × PBS. Mouse brains were sectioned into 30 μm slices, fixed with 4% PFA, washed in PBS and mounted for imaging as described above.

### Electrophysiology

Acute brain slices were obtained from wild-type (WT) and PD transgenic (TG) mice at postnatal day 10 (WT, n = 4 and TG, n = 4). The mice were deeply euthanized with CO_2_ followed by decapitation. Subsequently, the brains were swiftly extracted and placed in an artificial cerebrospinal fluid (aCSF) solution with the following composition:119 mM NaCl, 2.5 mM KCl, 26 mM NaHCO_3_, 1.25 mM NaH_2_PO_4_, 20 mM glucose, 2 mM CaCl_2_, 1 mM MgSO_4_, 0.4 mM ascorbic acid and 2 mM pyruvic acid. Coronal brain slices (300 μm) were prepared using a vibratome (Leica, Germany) and incubated at 32 °C for a minimum of 30 min. Throughout the preparation of acute slices, the solution was oxygenated using a mixture of 95% O_2_ and 5% CO_2_. The prepared acute hippocampal slices were transferred to a recording chamber filled with oxygenated aCSF containing 95% O_2_ and 5% CO_2_. The chamber maintained a continuous flow of this saturated aCSF. The slices were visualized using infrared differential interference contrast (IR-DIC) microscopy, using a BX51WI upright microscope (Olympus, Japan) equipped with a water-immersed 40 × objective lens (numerical aperture 0.8). Patch electrodes with tip resistances of approximately 4 MOhms were prepared using a pipette puller (Shutter Instrument, USA). The patch electrodes were filled with an internal solution containing the following concentrations: 20 mM KCl, 125 mM K-gluconate, 10 mM HEPES, 4 mM NaCl, 0.5 mM EGTA, 4 mM ATP, 0.3 mM TrisGTP and 10 mM phosphocreatine. The pH of the solution was adjusted to 7.2, and the osmolality was approximately 290–300 mOsm.

### Statistical analysis

Statistical analyses were performed using GraphPad Prism 9.5.1 (GraphPad Software, Inc.). All values are presented as the mean ± standard error of the mean (SEM). All groups of data were subjected to the Shapiro–Wilk normality test. If the datasets did not pass the test, nonparametric statistics were used. To test for statistical significance between two groups, unpaired two-tailed Student's* t* tests or Mann‒Whitney *U* tests were used. For three groups, one-way ANOVA (Kruskal‒Wallis) followed by Dunn’s multiple comparisions test was used. Two-way ANOVA followed by Bonferroni’s multiple comparisons test was used to analyze c-fos expression after whisker stimulation. Statistical significance was considered as follows: *p < 0.05, **p < 0.01.

## Results

### Underdevelopment of the cerebrovascular network in the familial PD mouse model

**Figure 1:**
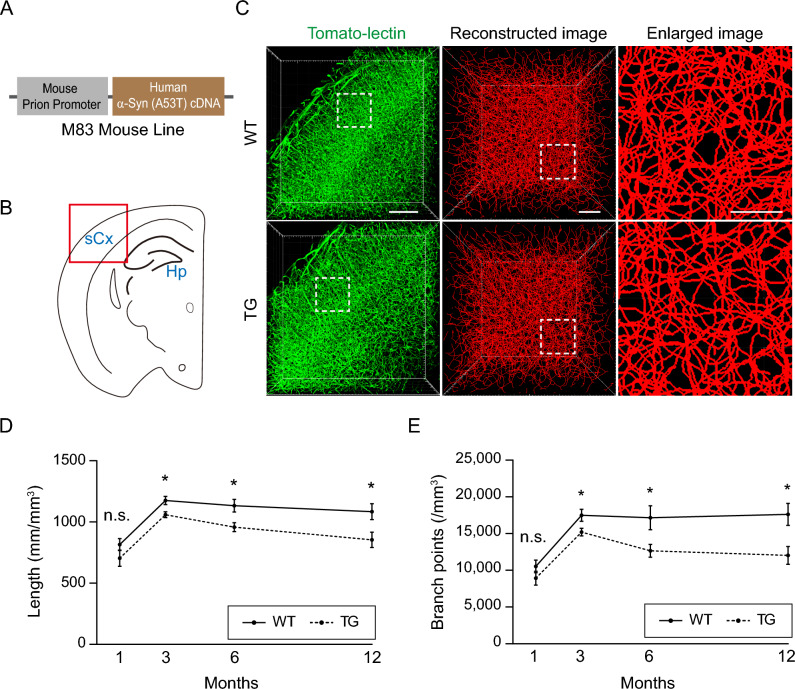
A young familial PD mouse model shows reduced cerebrovascular patterning. **(A)** Schematic of the M83 TG mouse line^17^. **(B)** Schematic drawing of a coronal view of the mouse brain. Red box indicates the imaged region. Hp, Hippocampus; sCx, somatosensory cortex. **(C)** Representative cleared 3D vessel (left) in the somatosensory cortex region and its reconstructed image (middle) after tomato-lectin labeling from 3-month-old homozygous M83 TG mice and littermate WT controls. The reconstructed images are obtained from the box area of the tomato-lectin staining. The right panels show a magnified view of the dotted box region in the reconstructed images. Scale bars from left to right panels = 500 μm, 100 μm and 50 μm. **(D-E)** Quantification of length (D), and branch points (E) of 3-D images from 1- (WT, n = 5, TG, n = 6), 3- (WT, n = 10, TG, n = 11; length, *p* = 0.0101; branch points, *p* = 0.0276), 6- (WT, n = 5, TG, n = 5; length, *p* = 0.0239; branch points, *p* = 0.0415) and 12- (WT, n = 4, TG, n = 5; length, *p* = 0.0416; branch points, *p* = 0.0215) month-old mouse brains in a mm^3^ volume. n.s. = not significant. The ‘n’ in each parenthesis indicates the total number of animals used for brain imaging. Four random cortical regions in each mouse brain were selected and analyzed for average values. Statical significances are analyzed between WT vs. TG groups for each month. Data are shown as the mean ± SEM. *p < 0.05; unpaired two-tailed Student's *t* tests.

To investigate whether α-Syn overexpression affects the overall structural defects of the brain vasculature, we performed three-dimensional (3-D) vascular imaging using the M83 PD transgenic (TG) mouse model that selectively expresses an A53T familial mutant form of human α-Syn in neuronal cells (Fig. 1A–C). The M83 mice were backcrossed to C57BL/6 J mice for more than 10 generations to minimize interindividual phenotypic variation caused by the genetic background. The 3-D analysis of the somatosensory cortex region (Fig. 1B) after tissue clearing allowed us to obtain a better image resolution and precise vessel analysis in many parameters, such as length and branch points. To our surprise, we found that 3-month-old homozygous PD mice exhibited less developed network formation in all parameters of the vascular analysis when compared to age-matched wild-type (WT) mice, much earlier than the onset of obvious motor symptoms (Fig. 1D, E). This reduction in vascularization remained consistent until 12 months of age. Given the common observation that relatively late-stage PD patients show a reduction in vessel density^10,11^, our findings occurred very early in M83 mice. While such defects were not significant in 1-month-old PD mice, a similarly reduced vascular phenotype was still observed.

To investigate whether the observed vascular defects in the 3-month-old PD mouse model were due to developmental delay, we measured cortical thickness and body weight, but neither showed significant alterations when compared to WT controls (Supplementary Fig. S1A-C). Furthermore, we examined whether overexpressed α-Syn induces vascular cell death near blood vessels, resulting in a reduction in cerebrovasculature, by analyzing the level of apoptotic cells through cleaved caspase-3 (CC3) immunostaining. No clear cell death in either 3-month-old WT or PD mice was detected. However, given previous reports that the M83 TG model exhibits functional vascular defects and motor deficits at approximately 8 months of age^17,28^, and our observation of a slight cerebrovascular reduction extending up to 12 months of age, we sought to investigate whether aged PD mice display vascular degeneration. Interestingly, we observed very few CC3-positive dying cells in 1-year-old PD mice, and there was no difference compared to the age-matched WT control (Supplementary Fig. S1D). These data suggest that the reduced vasculature may not be caused by direct vascular degeneration in the M83 TG model.

Since a previous report shows BBB defects in the 8-month-old M83 mouse model^28^, we also examined functional and structural changes in the BBB in young PD mice. However, we did not find any signs of BBB alteration such as changes in vascular permeability or tight junction protein levels in 3-month-old PD mice (Supplementary Fig. S2). This inconsistency is likely due to the age difference, as the 8-month-old mice began to show motor defects while the 3-month-old mice did not^28^. These results indicate that vascular network defects occur earlier during pathogenesis in the M83 PD model, as evidenced by the detectable neuronal symptoms in 8-month-old homozygous mice^17^.

### In young PD mice, the pathological form of human α-Syn is selectively expressed in neurons

**Figure 2:**
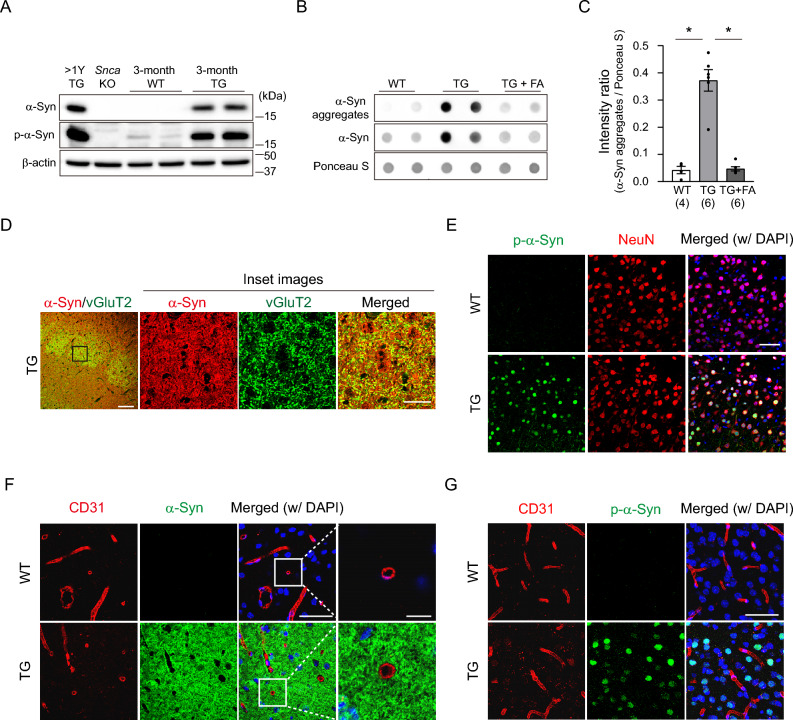
In young PD mice, the phosphorylated form of human α-Syn is significantly observed in neurons but not in blood vessels. **(A)** Western blot data showing the expression of α-Syn and its phosphorylated form (serine 129). The human α-Syn-specific antibody detects overexpressed human A53T α-Syn from M83 mice but not from WT or *Snca* knockout (KO) mice. The phosphorylated α-Syn-specific antibody also partially recognizes endogenous mouse α-Syn in WT mice. **(B)** Dot blot assay using a specific antibody that detects the human α-Syn aggregate (top) and an antibody that detects both human and mouse α-Syn (middle). Uncropped blot images are included in a Supplementary Information file. **(C)** Quantitative analysis of dot blot images. The intensity of human aggregated α-Syn expression is normalized to that of Ponceau S staining (bottom). Sample numbers are shown in parentheses below the graph. Data are shown as the mean ± SEM. One-way ANOVA (Kruskal‒Wallis test) followed by Dunn’s multiple comparisions test (WT vs. TG, *p* = 0.0277; TG vs. TG + FA, *p* = 0.0108). *p < 0.05. FA = formic acid. **(D)** Representative images of immunostaining for vGluT2 (green), a neuronal marker in the barrel cortex, and human α-Syn (red) in 3-month-old M83 mice. The inset with a black box shows high-resolution images. Note that both stainings are highly overlapping in the barrel cortex region. Scale bar = 100 μm; high-resolution images, scale bar = 25 μm. **(E)** Representative images showing immunostaining of phosphorylated α-Syn (green) and NeuN (red; neuronal marker). Note that most of the phosphorylated α-Syn is present in the neuron, particularly in the nucleus. Scale bar = 50 μm. **(F)** Representative images of immunostaining for CD31 (red), an endothelial cell marker, and human α-Syn (green) in 3-month-old WT and M83 mice. The insets with white boxes show high-resolution images. Note that human α-Syn is not detected in blood vessels. Scale bar = 50 μm; high-resolution images, scale bar = 10 μm. **(G)** Representative images showing immunostaining of phosphorylated α-Syn (green) and CD31 (red). Scale bar = 50 μm.

To investigate the potential link between the observed vascular underdevelopment in young PD mice and α-Syn toxicity, we evaluated the levels of both overexpressed human α-Syn and phosphorylated α-Syn (Ser129), a known toxic form, in 3-month-old PD mice. As shown in Fig. 2A, human α-Syn exhibited selective and significant expression in the 3-month-old PD mouse brain, in contrast to WT or *Snca* knockout. In addition, phosphorylated α-Syn was also highly expressed in the brains of both young and old PD mice compared to the WT control, correlating with the overall increase in total human α-Syn. To further examine whether aggregated α-Syn, a hallmark of PD pathogenesis, is generated in 3-month-old PD mice, we conducted dot blot analysis using a specific antibody that detects α-Syn aggregates (Fig. 2B, C). We found a significantly higher level of aggregated α-Syn in PD mice than that in WT controls. Moreover, treatment with 50% formic acid, a protein denaturation solution, resulted in barely detectable levels of the aggregated form confirming that the 3-month-old young PD mice produced aggregated α-Syn, as well as the phosphorylated form. We also observed a reduction in the total levels of α-Syn in the formic acid-treated TG samples, possibly attributable to the antibody’s inefficiency in recognizing denatured α-Syn aggregates.

Next, to test the possibility that the overexpressed toxic form of α-Syn directly causes vascular defects through neuron-to-endothelial cell α-Syn transmission^29–31^, we conducted a thorough examination of its localization in the brain tissue of young PD mice. Overexpressed human α-Syn was predominantly expressed within vGluT2-positive neurons in the barrel cortex region, suggesting that human α-Syn is likely to be abundant in neurons at this age (Fig. 2D). Phosphorylated α-Syn was also primarily localized within NeuN-positive neurons, especially in the nucleus (Fig. 2E). However, despite the high level of α-Syn expression across brain tissues, we were unable to detect any human α-Syn or phosphorylated α-Syn in the vasculature (Fig. 2F, G). These results suggest that the overexpressed familial mutant form of human α-Syn may not directly affect the underdevelopment of the cerebrovasculature through neuron-to-endothelial cell transmission in the M83 model.

### Overexpressed human α-Syn does not induce abnormal inflammatory responses in young PD mice

**Figure 3:**
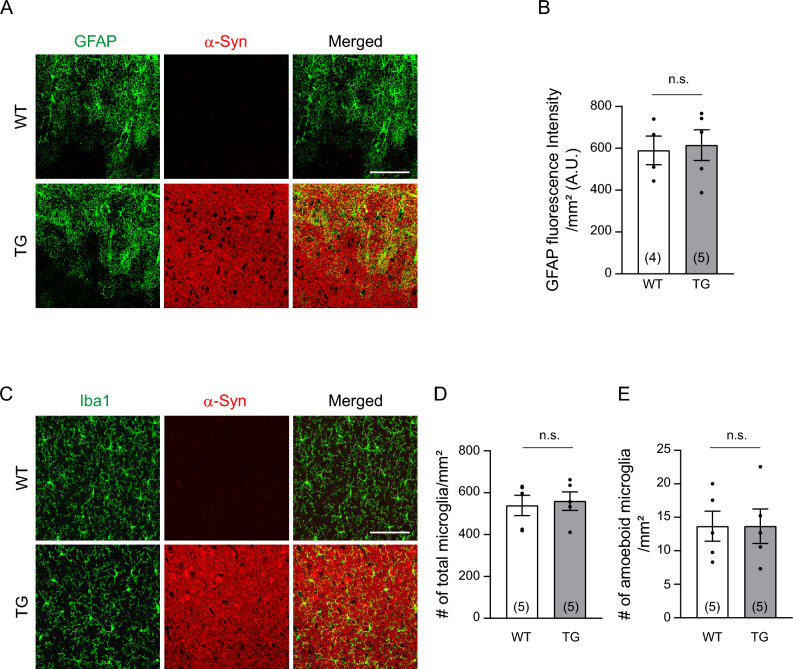
Young M83 TG mice do not induce an inflammatory response. **(A)** Representative coimmunostaining for glial fibrillary acidic protein (GFAP, green; a marker of activated astrocytes) and α-Syn (red) at 3 months of age. Scale bar = 100 μm. **(B)** Quantitative analysis of GFAP expression (green fluorescence intensity/mm^2^ of cortical area; Unpaired two-tailed Student’s *t* test, *p* = 0.8122). A.U., Arbitrary Unit **(C)** Representative coimmunostaining of Iba 1 (green; a marker for microglia) and α-Syn (red). Scale bar = 100 μm. **(D-E)** Quantification of total microglia number (D, Mann–Whitney test, *p* = 0.8413) and amoeboid form (E, Unpaired two-tailed Student’s *t* test, *p* = 0.9963) per mm^2^ of area. The total number of mouse brains analyzed is indicated in each set of graphs. Data are shown as the mean ± SEM. n.s. = not significant (P > 0.05).

Although we did not detect toxic α-Syn directly in contact with the vasculature, it is possible that some leaky α-Syn may be present in the perivascular area or may be taken up by other nonneuronal cells, indirectly affecting vascularization. As toxic α-Syn has been demonstrated to activate microglia and astrocytes^32,33^, we investigated whether abnormal inflammatory responses occurred in young PD mice. However, we found no significant changes in GFAP-positive active astrocytes and Iba1-positive microglia in the 3-month-old mice compared to WT control mice (Fig. 3). These results suggest that early neuronal expression of toxic α-Syn in the young M83 PD model interferes with the formation of the cerebrovascular network, rather than through direct vascular degeneration or abnormal inflammatory responses.

### Neonatal PD mice exhibit reduced angiogenic potential

**Figure 4:**
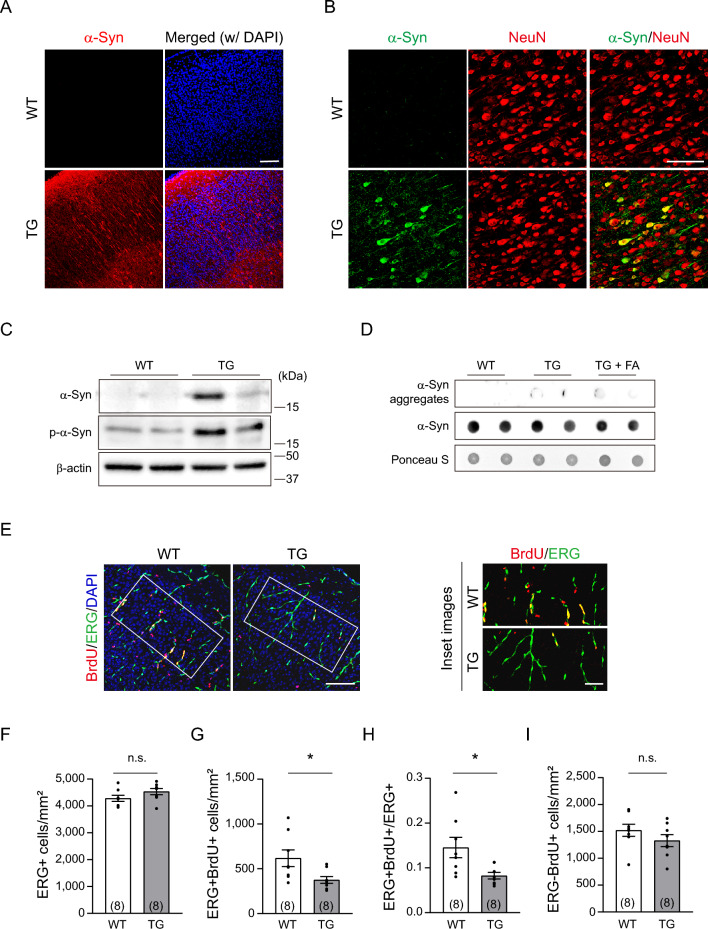
M83 TG mice show reduced endothelial cell proliferation at P10 in the presence of toxic α-Syn. **(A)** Representative images of human α-Syn expression (red) in the mouse cortex at P10. Scale bar = 100 μm. **(B)** Representative coimmunostaining images of human α-Syn expression (green) and NeuN (red) in the mouse cortex at P10. Scale bar = 100 μm. **(C)** Western blot images of human α-Syn and its phosphorylated form expression at P10. **(D)** Dot blot assay with P10 brain homogenates. Note that aggregated α-Syn has not yet clearly formed in M83 TG Mice. FA = formic acid. Uncropped blot images are included in a Supplementary Information file. **(E)** Representative images of immunostaining for ERG (green), an angiogenic endothelial cell marker, and BrdU (red). Magnified images of the white boxes are shown on the right. Scale bar = 100 μm; enlarged image, scale bar = 25 μm. **(F-I)** Quantification results of (E) in a mm^2^ boxed area. The total number of ERG-positive ECs (F, *p* = 0.1353), the ERG- and BrdU-double-positive ECs (G, *p* = 0.0301), the ratio of proliferating ECs (H, *p* = 0.0198) and the BrdU single-positive cells (I, *p* = 0.2466) are shown. The number of mouse brains analyzed is indicated in each set of graphs. Data are shown as the mean ± SEM. n.s. = not significant. *p < 0.05; unpaired two-tailed Student's *t* tests.

To examine the possibility of an abnormal angiogenic process contributing to the reduced cerebrovasculature in young PD mice, we evaluated the rate of endothelial proliferation. As the mouse developmental period exhibits two distinct waves of vigorous brain endothelial proliferation—one occurring during embryonic days E10.5-E15.5 and the other during postnatal days P5-P10^34^—we analyzed the proliferation rate at the end of the second wave, which was at P10. First, we investigated whether both human α-Syn and phosphorylated α-Syn are expressed in brains of P10 PD mice before proliferation analysis. As shown in Fig. 4A-C, P10 PD mice showed high levels of human α-Syn expression in neuronal cells throughout the brain with low levels of detectable toxic phosphorylated α-Syn. However, both normal and toxic forms of α-Syn exhibited interindividual variation in expression (Supplementary Fig S3A). Notably, a significant correlation was observed between the levels of each α-Syn form (r = 0.6162, *p* = 0.0084. Supplementary Fig S3B). Furthermore, aggregated α-Syn was not clearly detectable at P10 (Fig. 4D) when compared to 3-month-old PD mice, as shown in Fig. 2B. In contrast to the findings in 3-month-old TG mice (Fig. 2B), the total levels of α-Syn were not reduced even after 50% formic acid treatment. This observation suggests that the antibody designed to recognize all forms of α-Syn proteins may efficiently target the monomeric form of α-Syn. Taken together, these findings suggest that human α-Syn, along with phosphorylated forms, is expressed during the active angiogenic sprouting period in M83 PD mice.

To measure the proliferation rate specific to endothelial cells, we performed immunostaining with ERG (a marker for angiogenic endothelial cells)^35^ and BrdU incorporation (a marker for newly synthesized DNA) (Fig. 4E). Although the total number of ERG-positive cells was comparable between WT and PD mice, the number of ERG- and BrdU-double-positive cells was notably lower in PD mice (Fig. 4F–H). However, no significant difference was observed in the number of BrdU single-positive cells between WT and PD mice (Fig. 4I), suggesting that the proliferation potential of other types of brain cells may not be critically affected at this stage. These findings indicate that during the active angiogenesis period, PD mice have a lower number of proliferating endothelial cells. Furthermore, we observed no correlation between the expression levels of both forms of α-Syn and ERG- and BrdU-double-positive cells at P10 (Supplementary Fig. S3C). Taken together, these data suggest that certain levels of neuronal overexpression of human α-Syn may be sufficient to influence brain endothelial proliferation during the early neonatal period, impairing vascular network formation in the PD model.

### Overexpressed human α-Syn reduces neuronal activity in PD neonates

**Figure 5:**
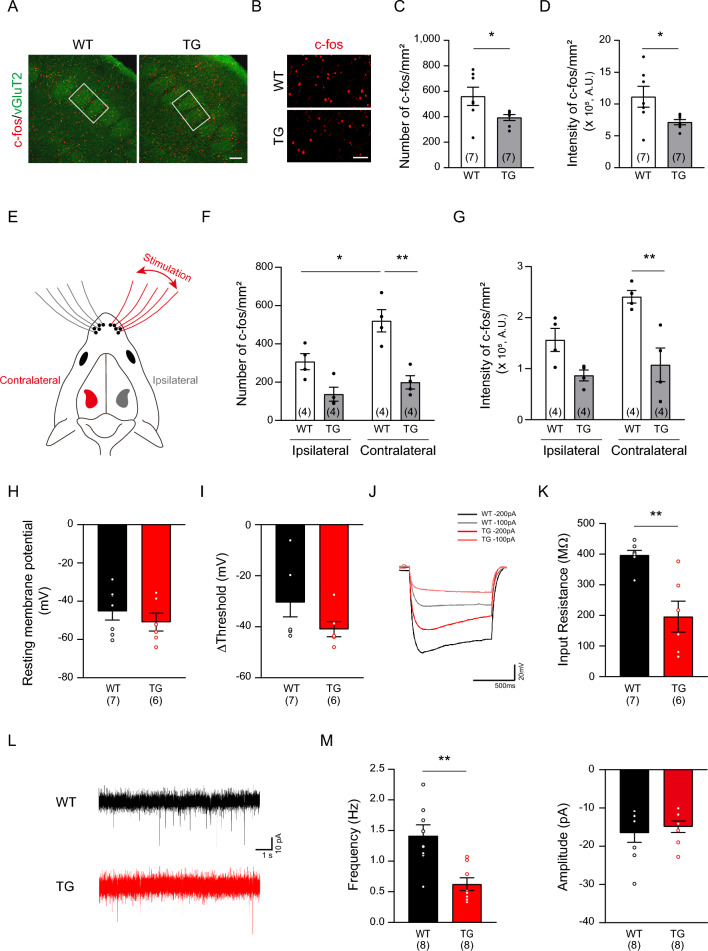
M83 TG mice show reduced neuronal activation in the P10 cortex. **(A)** Representative coimmunostaining of c-fos (red; an immediate early gene) and vGluT2 (green; a glutamatergic excitatory neuronal marker) at P10. **(B)** Magnified images from the white box areas of (A). Scale bar = 100 μm; enlarged image, scale bar = 25 μm. **(C-D)** Quantitative analyses of c-fos number (C, Mann–Whitney test, *p* = 0.0262) and intensity (D, Unpaired two-tailed Student’s *t* test, *p* = 0.0365) in mm^2^ of the somatosensory cortex. The total number of mouse brains analyzed is indicated in each set of graphs. (**E**) Schematics for whisker stimulation. **(F-G)** Quantification of c-fos number (F, *p* = 0.0297 in contralateral WT vs. ipsilateral WT; *p* = 0.0014 in contralateral WT vs. contralateral TG) and intensity (G, *p* = 0.0055 in contralateral WT vs. contralateral TG). The total number of mouse brains analyzed is indicated in each set of graphs. Data are shown as the mean ± SEM. *p < 0.05, **p < 0.01; two-way ANOVA with Bonferroni’s multiple comparisons test (F and G). **(H)** Intrinsic membrane properties of L4 neurons in the somatosensory cortex of the WT and PD models (-45.24 ± 4.62 mV in WT and -50.88 ± 4.67 mV in TG). (**I**) Voltage difference between resting membrane potential and threshold for action potential (-30.46 ± 5.65 mV in WT and -40.96 ± 2.96 mV in TG, *p* = 0.10041). (**J**) Representative traces of voltage responses upon hyperpolarizing current injections. Note that the same amount of hyperpolarizing current injection caused smaller voltage changes in the TG. **(K)** Quantification of input resistance (396.80 ± 15.64 MOhm in WT and 195.90 ± 50.69 MOhm in TG, *p* = 0.00534). **(L)** Representative traces of spontaneous excitatory synaptic currents (sEPSC). **(M)** Quantification of frequency (Left graph, 1.41 ± 0.18 Hz in WT and 0.62 ± 0.11 Hz in TG, *p* = 0.00276). Quantification of amplitude (Right graph, 16.55 ± 2.44 pA in WT vs. 14.92 ± 1.50 pA in TG). Scale bar = 1 s and 10 pA. The total number of cells patched from 4 WT and 4 TG animals is shown in each graph set (H, I, K, and M). Data are shown as the mean ± SEM. **p < 0.01; unpaired two-tailed Student's *t* tests.

Previous studies have shown that during the critical period of sensory neuronal plasticity, there is a neurovascular interaction whereby neuronal activity regulates the brain vasculature^21^. Thus, abnormal α-Syn accumulation leading to insufficient neuronal activation may interfere with the formation of the cerebrovascular network in young PD mice. To test whether vascular abnormalities correlate with reduced neuronal activation in the barrel cortex, we performed immunostaining with c-fos, an immediate early gene that responds to changes in neuronal activity^36^. As shown in Fig. 5A–D, both the number and intensity of c-fos-positive cells were significantly lower under normal conditions in PD mice than those in WT mice at P10. Interestingly, whisker stimulation led to a significant increase in c-fos-positive cells in the contralateral hemisphere of WT mice compared to the ipsilateral hemisphere (Fig. 5E–G). In contrast, PD mice failed to exhibit an increase in c-fos-positive cells in the stimulated barrel cortex, indicating that reduced neuronal activation in PD mice hampers the formation of the brain vasculature (Fig. 5E–G). To further confirm the reduced circuit activity and to elucidate the cause of the reduced neuronal activation, we measured the neuronal excitability with whole-cell patch clamp recording from layer 4 excitatory neurons of wS1. The resting membrane potential, threshold and the voltage difference between the two voltages (ΔThreshold) were not significantly altered (Fig. 5H–I). However, the input resistance representing the magnitude of voltage changes in response to the same current injection was significantly reduced in the PD model (Fig. 5J,K). Because reduced excitability of cortical neurons could lead to reduced network activity, we analyzed the spontaneous excitatory postsynaptic current (sEPSC). Corroborating the reduced c-fos labeling and excitability of the neurons, we found a significant reduction in the frequency of sESCS (Fig. 5L,M). On the other hand, we did not detect any changes in the amplitude of sEPSCs, suggesting that postsynaptic strength cannot be the main source of reduced circuit activity. Taken all together, these results suggest that even at the early stage of PD progression, neocortical sensory processing was downregulated in the M83 TG model.

### Activity-dependent angiogenic gene expression in the endothelial cells is altered in young PD mice

**Figure 6:**
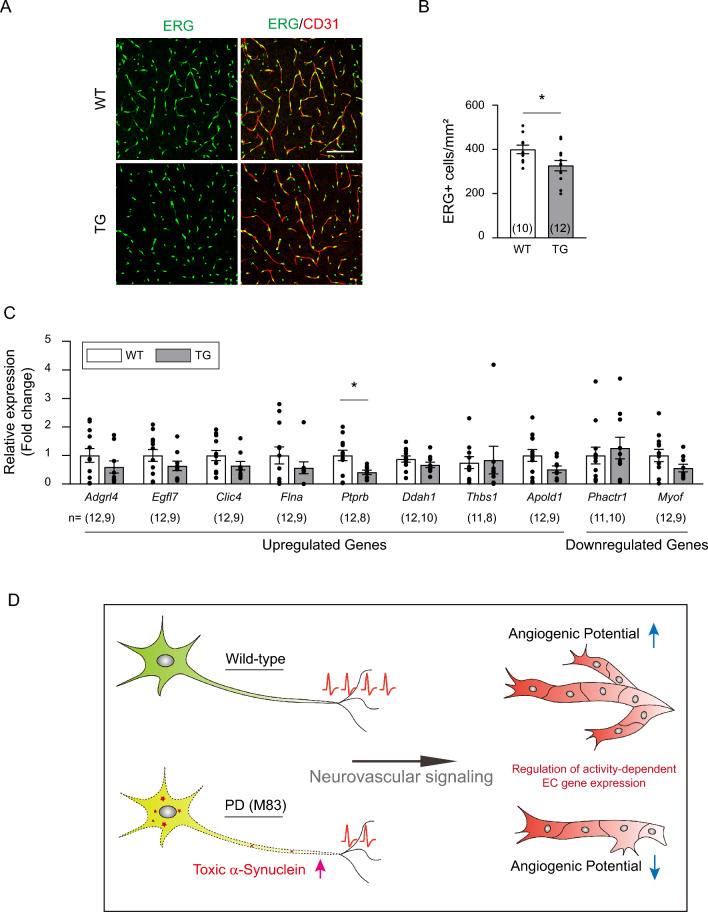
Expression patterns of activity-dependent angiogenic genes are altered in 1-month-old PD mice. (A) Representative coimmunostaining of ERG (green) and CD31 (red) in 1-month-old mice. (**B**) Quantification of ERG-positive cell numbers in mm^2^ of the somatosensory cortex. The total number of mouse brains analyzed is indicated in each set of graphs. **(C)** Quantitative analysis of gene expression patterns of activity-dependent angiogenic genes from isolated capillaries of the somatosensory cortex in 1-month-old mice using RT‒qPCR (*Ptprb*, Unpaired two-tailed Student’s *t* test, *p* = 0.0215). The number of mouse brains analyzed is indicated at the bottom of each set of graphs. Data are shown as the mean ± SEM. *p < 0.05; unpaired two-tailed Student's *t* tests or nonparametric Mann‒Whitney test was performed. **(D)** Graphical summary of how young M83 mice exhibit underdeveloped cerebrovascular patterning through abnormal neuronal activation.

Previous studies have profiled gene expression in endothelial cells under different neuronal activations^37,38^. To test if young PD mice show any alterations in the gene expression in brain ECs in response to neuronal activity, we selected a group of candidate genes that are involved in angiogenesis, including 8 upregulated and 2 downregulated genes (Fig. 6C and Supplementary Table S1). Initially, we analyzed multiple genes in PD mice at P10, but we did not observe any significant changes in their expression (Supplementary Fig. S4), possibly because of the considerable individual variation shown in Supplementary Fig. 3A. To minimize this variability, we examined the gene expression pattern in 1-month-old mice, as vascular formation is relatively stable at this stage, although a small population of proliferating endothelial cells still persists^39^. We first examined whether the angiogenic potential was similarly reduced at this stage, as observed at P10 (compared to Fig. 4E–4H). In contrast to the substantial presence of positive non-endothelial cells in the area between the cortex and hippocampus (Supplementary Fig. S5), we found a very limited number of BrdU-positive endothelial cells in the barrel cortex, consistent with a previous study^39^. In contrast to BrdU staining, ERG-positive cells were evident in the cortex of 1-month-old mice (Fig. 6A), albeit at a tenfold lower number than that observed in the P10 cortex (compared to Fig. 4F). At 1 month of age, there was a slight but statistically significant decrease in the number of ERG-positive cells in the barrel cortex of PD mice when compared to the WT control (Fig. 6B). This suggests that the impaired angiogenic potential persisted until at least 1 month of age.

The analyses of changes in neuronal activity-dependent endothelial gene expression showed more apparent changes in the anticipated gene expression patterns at 1 month of age. Of the genes analyzed, 7/8 genes that were expected to be upregulated by neuronal activation showed decreased expression, while 1/2 genes that were expected to be downregulated exhibited an increased expression pattern in young PD mice (Fig. 6C). Among the genes analyzed, the only one that displayed statistical significance was *Ptprb*, a vascular endothelial protein tyrosine phosphatase that interacts with VE-cadherin, Tie-2, and VEGFR2 proteins to regulate vascular remodeling^40^. While we did not observe definitive changes in the tested target genes at 1 month of age, these findings imply that compromised neuronal activation in young PD mice may ultimately lead to reduced vasculature in adulthood, possibly through alterations in angiogenic gene expression (Fig. 6D).

## Discussion

Accumulating evidence suggests that various cerebrovascular impairments are major predisposing factors in the development of many neurodegenerative diseases. A recent multifactorial data-driven analysis that utilized brain imaging and biomarkers reports that vascular dysfunction is an early event that precedes amyloid-beta (Aβ) deposition, a hallmark of late-onset Alzheimer’s disease progression^41^. Likewise, studies on PD have focused on deciphering early vascular alterations as key etiologic factors that drive disease progression, characterized by the aggregation of α-Syn and resultant neurotoxicity. Because clinical symptoms of PD generally manifest in older individuals and postmortem specimens are typically the only available source for pathologic analysis, including that of vascular changes, there are inherent limitations in studying the full spectrum of disease progression from early to late stages in human subjects. Despite these limitations, postmortem studies of PD have revealed various manifestations of vascular damage, such as increased permeability of the BBB^5^, pathological angiogenesis^42^ and vascular degeneration^43^. These vascular defects are most likely caused by the direct effect of toxic α-Syn accumulation on endothelial cells or pericytes during the relatively late stage of PD progression. However, our study reveals a new pathological mechanism that toxic α-Syn expression in neurons indirectly affects vascular pattering during the early phase of PD.

Numerous animal models have been generated to replicate human PD, which have demonstrated vascular changes that closely mirror those seen in disease progression. In the human α-Syn overexpression mouse model, long-term analysis reveals stepwise pathological events that included pericyte activation, BBB disruption, and morphological changes from angiogenesis to degeneration^44^. Notably, a previous investigation of the M83 PD model demonstrates a decrease in tight junction proteins, increased BBB permeability, and astrocytic activation in 8-month-old PD mice, coinciding with the accumulation of oligomeric α-Syn, whereas only a minor decline in occludin is observed in younger PD mice at 4 months of age^28^. Although aggregated α-Syn was present, our findings did not provide any evidence of BBB leakage or inflammatory activation in 3-month-old M83 PD mice (Fig. 3 and Supplementary Fig. S2). Instead, we found a slight yet significant reduction in brain vasculature formation in these young mice through 3-D analysis (Fig. 1), a method not seen in previous studies, which mostly relied on 2-D immunostaining. Many transgenic mouse models with genetically modified α-Syn genes are currently available, displaying diverse histological and behavioral phenotypes at varying ages of disease onset^16^. None of these mice have shown an underdeveloped cerebrovasculature due to abnormal angiogenesis at a very early stage of PD. This suggests that such defects might be overlooked since they occur far from the critical period of disease progression and their phenotypic changes are not dramatic. At present, it remains unclear whether the mild reduction in vascular patterning is directly linked to PD pathology or has any role in abnormal brain function. However, since the brain microvasculature is essential for the supply of oxygen and nutrients to neurons within a certain distance^45^, it is plausible that underdeveloped vessels could impede normal brain function throughout the lifespan. In line with this hypothesis, we found reduced sensory stimulation-driven cortical activity and excitability in the M83 mice even in the very early stage of development (Fig. 5H–M).

In contrast to previous findings that show the presence of toxic α-Syn in endothelial cells resulting in vascular pathology^44^, we did not observe any conclusive evidence of toxic α-Syn in the M83 vasculature during the early stage of PD (Fig. 2F,G). This discrepancy could account for the varied degree of pathology seen across different transgenic models, as well as their differences in timing of disease onset. Instead, impaired vascular network formation in M83 PD mice may be due to the accumulation of phosphorylated α-Syn and filamentous aggregates in neurons that disrupt activity-dependent angiogenic processes. These processes are well documented in postnatal cerebrovascular development through sensory stimulation^21^. Consistent with these previous findings, recent studies employing next-generation sequencing have identified potential mechanistic factors that regulate activity-dependent cerebrovascular plasticity^37,38^, including those associated with vascular patterning. However, presumably due to the variability among individual mice at 1 month of age, we were unable to detect a statistically significant positive correlation among all candidate gene expression changes between PD mice and their littermate controls. Since there was only a mild vascular reduction (approximately 10%) in young PD mice (Fig. 1), it is probable that detecting clear differences in specific gene expression would be challenging. Nevertheless, most of the candidate genes known to be upregulated in activity-dependent angiogenesis tended to be reduced in the 1-month-old PD mice (Fig. 6C) when compared to the expression in P10 PD mice (Supplementary Fig. S4). This suggests that the accumulated inappropriate endothelial gene expression is likely involved in the decreased vascular network.

Although dynamic vascular sprouting and pruning occur during the first postnatal month, after which microvessel formation stabilizes in mice, it is known that certain vascular changes persist throughout life in response to metabolic demands^39^. Notably, vascular remodeling is especially critical following severe vascular insults such as chronic hypoxia in the brain. Patients with PD have shown a high risk of stroke, while those with a history of stroke have exhibited, in turn, an increased risk of PD^3,46,47^. In rodent stroke studies, ischemia trigger sustained α-Syn aggregation, resulting in neuronal loss in the PD model, and prevention of poststroke α-Syn expression shows neuroprotective outcomes^48,49^. Despite these pathologically relevant findings, the mechanistic relationship between vascular remodeling and α-Syn expression remains elusive. A previous study demonstrates that the enhancement of neuronal activity promotes angiogenesis in the sensory cortex penumbra of ischemic adult mice^50^. This highlights the critical importance of regulating neuronal activity effectively to reconstruct damaged vasculature.

Because of the complex causal relationships in neurovascular interactions, it is not feasible to approach the two systems separately in terms of pathophysiology. The current prevailing view of neurodegenerative diseases such as PD suggests that various vascular pathologies precede significant disease onset. However, our study reveals that in the M83 familial PD mouse model, vascular patterning defects begin with the downregulation of neuronal activity (Fig. 5). To our knowledge, our findings introduce an important concept: the abnormal accumulation of toxic α-Syn within neurons and subsequent neuronal dysfunction during the early stages of PD may indirectly impact vascular patterning and ultimately trigger worsening cycles of neurovascular interaction, contributing to disease progression. This suggests that even minor intraneuronal abnormalities may have significant consequences for neurovascular health in PD. Although we have discovered the mechanistic link in the familial PD mouse model, it would be interesting to explore whether a similar mechanism exists in the majority of sporadic PD cases and other synucleinopathies, particularly in young postmortem specimens. Furthermore, since cerebrovascular angiogenesis involves multiple cell types, such as pericytes, glia and immune cells, in addition to endothelial cells, it would be intriguing to further investigate how each cell type is affected by toxic α-Syn during the early stages of PD progression.

### Supplementary Information

Supplementary Information.

## Data Availability

The datasets used and/or analyzed during the current study are available from the corresponding author upon reasonable request.
