# Supplementary material for: Impaired neuronal activity as a potential factor contributing to the underdeveloped cerebrovasculature in a young Parkinson’s disease mouse model
Source: Sci Rep. 2023 Dec 18;13:22613. doi: 10.1038/s41598-023-49900-w (PMC10730707; doi:10.1038/s41598-023-49900-w)
Supplement: Supplementary file 1 — Supplementary Information. [file 41598_2023_49900_MOESM1_ESM.pdf]

Supplementary Material

Impaired neuronal activity as a potential factor contributing to the underdeveloped cerebrovasculature in a young Parkinson's disease mouse model

Jin-Young Jeong[1,3], Hyun Jung Lee [2], Namsuk Kim[1], Yan Li[1], Jong-Cheol Rah[2] and Won-Jong Oh[1,*]

[1]Neurovascular Biology Laboratory, Neurovascular Unit Research Group, Korea Brain Research Institute, Daegu 41062, South Korea

[2]Sensory and Motor System Research Group, Korea Brain Research Institute, Daegu 41062, South Korea

[3]Department of Brain Sciences, Daegu Gyeongbuk Institute of Science and Technology, Daegu 42988, South Korea

*Corresponding author

E-mail: ohwj@kbri.re.kr (WO)

Supplementary figures

Supplementary table

Uncropped blot images

Supplementary Figure 1

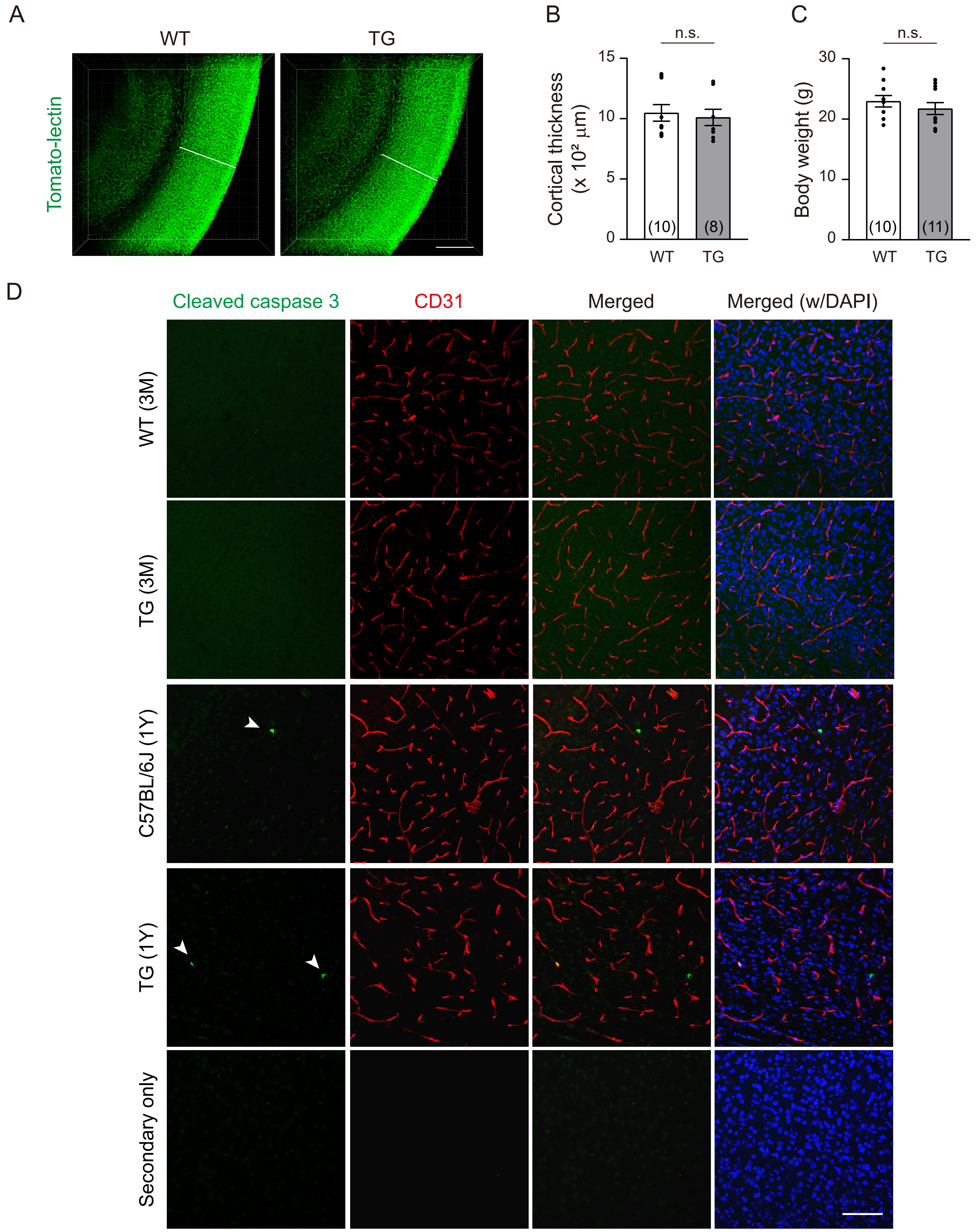

Supplementary Fig 1. Young M83 TG mice exhibit normal growth rates and do not show significant cell death. **(A)** Representative images of cortical area for thickness analysis of 3-month-old mice. Scale bar = 500 μm. **(B)** Quantification of cortical thickness from (A) (p = 0.5726). **(C)** Comparison of body weight in WT and M83 TG mice (p = 0.3763). Data are shown as the mean ± SEM. n.s. = not significant. Mann-Whitney test. The total number of animals is indicated in each set of graphs (B-C). **(D)** Representative images of cell death analysis by cleaved caspase-3 (CC3) immunostaining. No dead cells were observed in the 3-month-old WT and TG mice. A few CC3-positive cells were detected (white arrowheads) in both WT and TG mice at 1 year of age, in contrast to the negative control using the secondary antibody alone. Scale bar = 100 μm.

Supplementary Figure 2

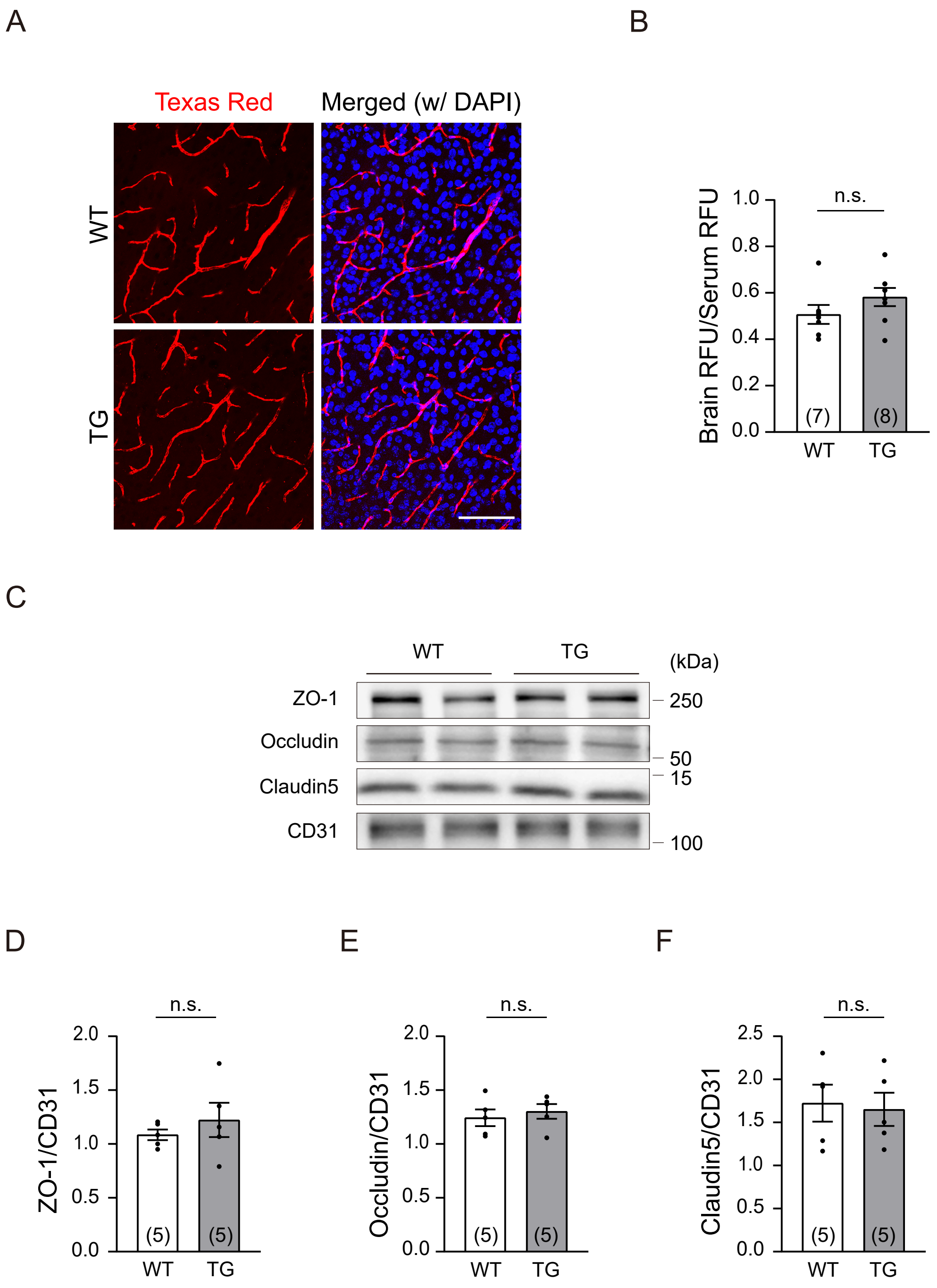

Supplementary Fig 2. Normal BBB function and structure in young PD mice. **(A)** Representative brain images to analyze BBB leakiness after circulating 10 kDa fluorescent dye. Scale bar = 100 μm. **(B)** Permeability index analysis of mice injected with a 1 kDa fluorescent dye (p = 0.2082). RFU = raw fluorescence units. **(C-F)** Representative images of Western blot data showing the expression of tight junction proteins and their quantification (ZO-1/CD31, p = 0.4322; occludin/CD31, p = 0.5872; claudin5/CD31, p = 0.8079). Uncropped blot images are included in a Supplementary Information file. Data are shown as the mean ± SEM. n.s. = not significant. Unpaired two-tailed Student's t tests. The total number of animals for analysis is indicated in each set of graphs

Supplementary Figure 3

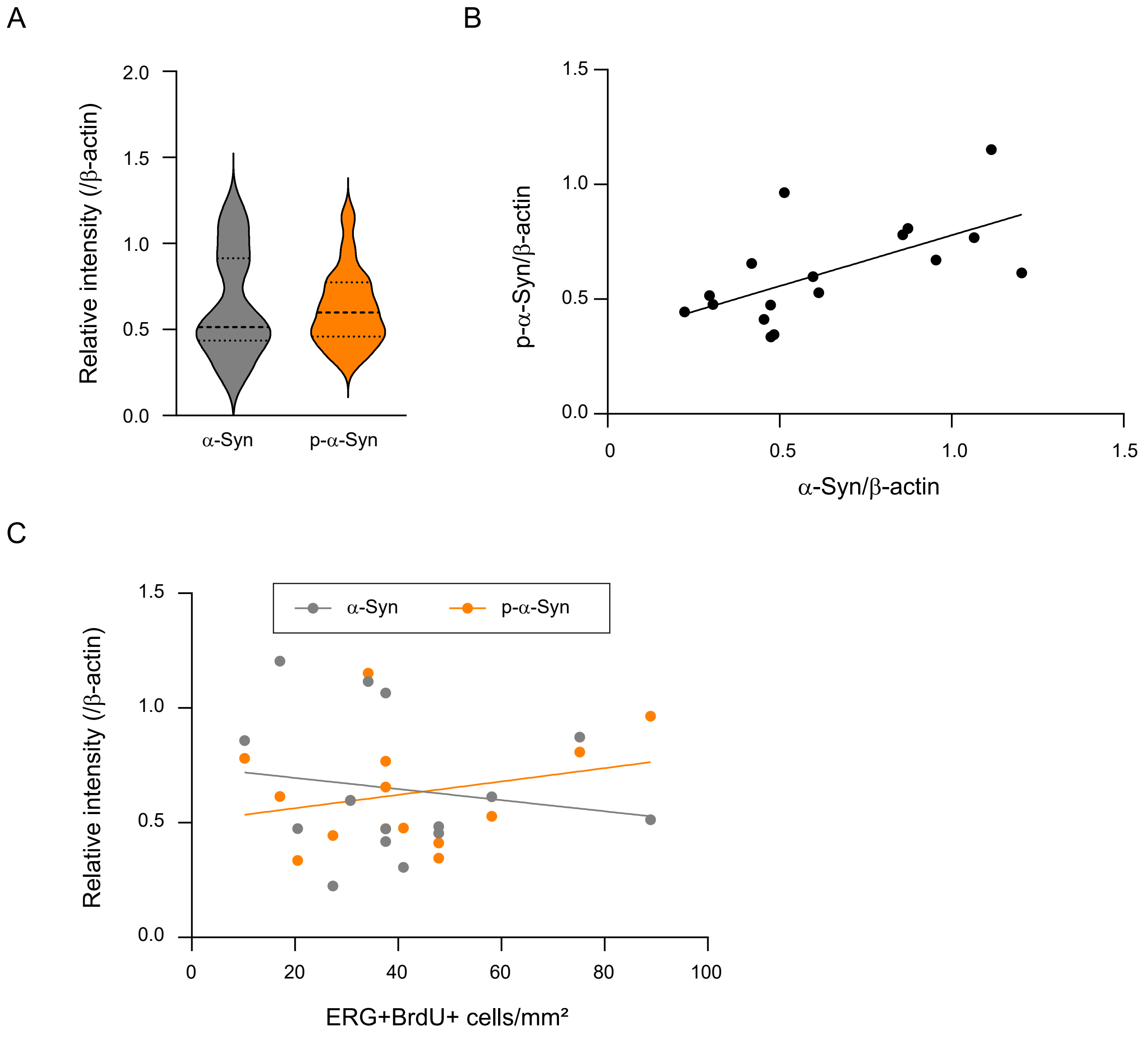

Supplementary Fig 3. Neonatal PD mice exhibit variable levels of α-Syn expression and these levels do not correlate with endothelial cell proliferation. **(A)** Variability of overexpressed human α-Syn (gray) and phosphorylated α-Syn (orange) expression patterns (n = 17 animals). **(B)** Correlation curve of human α-Syn and phosphorylated α-Syn (orange) expression levels (r = 0.6162, *p* = 0.0084, n = 17 animals). **(C)** Correlation curve of human α-Syn or phosphorylated α-Syn vs. the number of ERG and BrdU double positive cells (α-Syn, r = -0.1674, *p* = 0.5509, n = 15 animals; p-α-Syn, r = 0.2607, *p* = 0.348, n = 15 animals).

Supplementary Figure 4

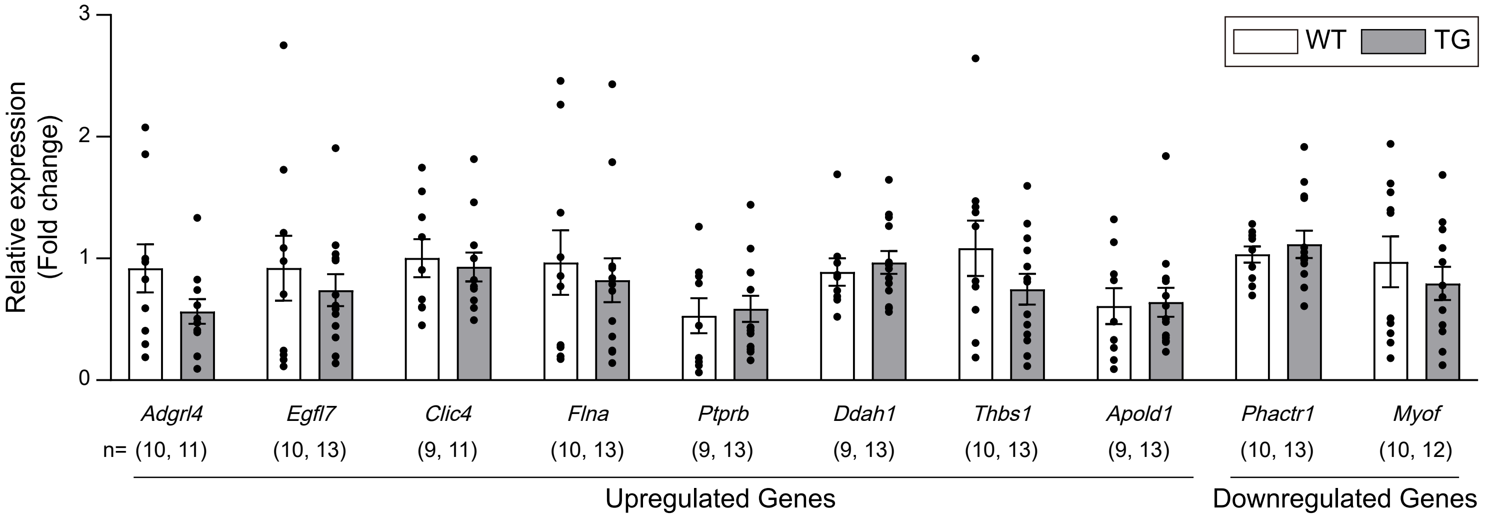

Supplementary Fig 4. Neonatal PD mice exhibit a high degree of variability in the angiogenic gene expression pattern. Quantitative analysis of gene expression patterns of activity-dependent angiogenic genes from isolated capillaries of somatosensory cortex in P10 mice using RT–qPCR. The number of mouse brains analyzed is indicated at the bottom of each set of graphs. Data are shown as the mean ± SEM. All data were not statistically significant; unpaired two-tailed Student's t tests or nonparametric Mann–Whitney test was performed.

Supplementary Figure 5

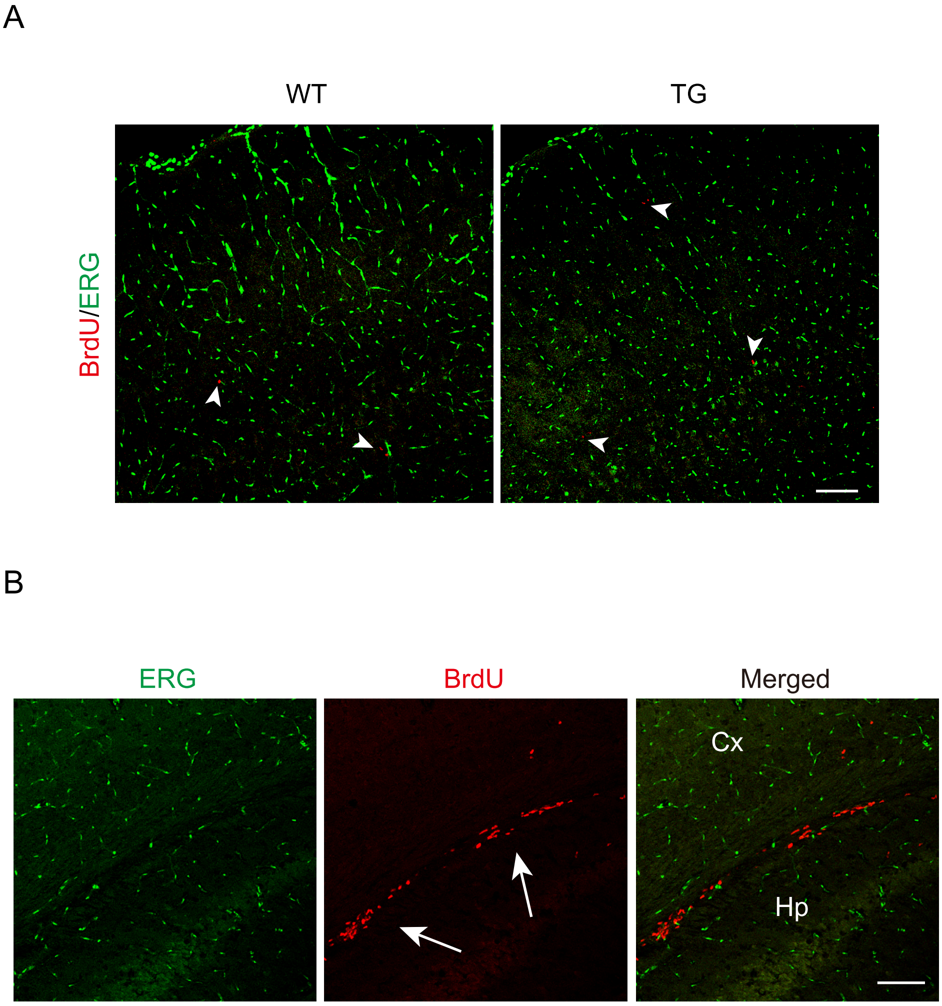

Supplementary Fig 5. Reduced angiogenic potentials in 1-month-old mice. (A) Representative somatosensory cortical area images of coimmunostaing of ERG (green) and BrdU (red). Very few BrdU-positive cells are observed (white arrowheads) Scale bar = 100 μm. (B) BrdU-positive cells (arrows) between cortex (Cx) and hippocampus (Hp) regions.

Table S1. Primer sequence information for RT-qPCR

Gene	Forward primer	Reverse primer
Adgrl4	GTGAAATCCAAAGCACCAGGAC	CCAGCAATGATAGAGCAGACCA
Egfl7	AGACCCAGCCGTAGAGTGTG	TCCGGTAGATGGTTCGGTAG
Clic4	AAGGAATGACAGGCATCTGGAG	TGGTAAGTCTCTTGGCGACATC
Ddah1	AGGGCGAGGAGGTGGATTTC	ACGTCCTCCACGAACACGCA
Flna	CGAGTATGCTGTTCATGTGCTG	CACACCAGTCTTCTCCAATCCA
Thbs1	TCCGAGTTGCAAAGGGAGATG	TAAGAAGGACGTTGGTAGCTGA
Myof	TATATGACTGGGACCGGCTTAC	AGCTCCCGAGGATGAGAAATC
Phactr1	AGAAGCGGGAAATCAAGAGGAG	CTTCGGTCATAGTCTTGGGCAT
Ptprb	AACGGCCTTGTGGATAACACT	TAGACGCTGACAAGCTAGAGAC
Apold1	CTTCATCGTCTTCTTCGGCTCA	TCCAGGCTCTCTGACAGTTTCT
Gapdh	TGACGTGCCGCCTGGAGAAAC	CCGGCATCGAAGGTGGAAGAG

Uncropped blot images

Full-length western blot images

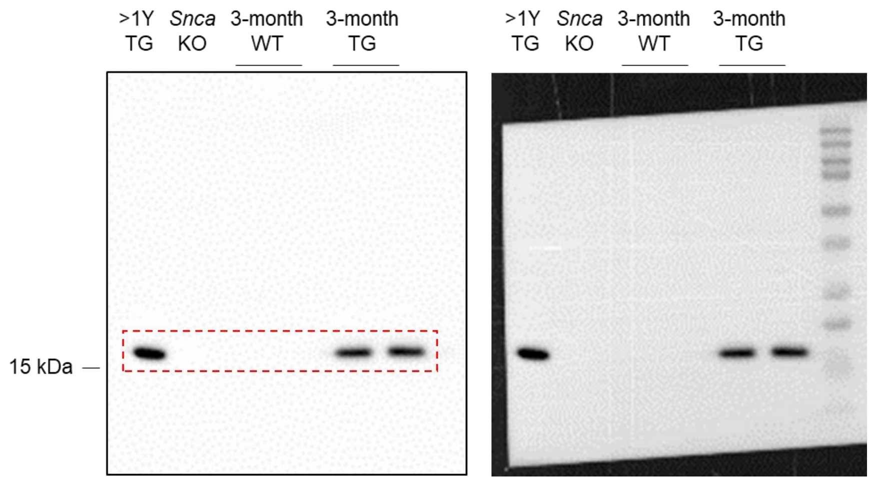

Western blot image for Fig. 2A. Full-length blot image with anti-α-Syn before cropping on the left and merged image with protein size marker on the right side.

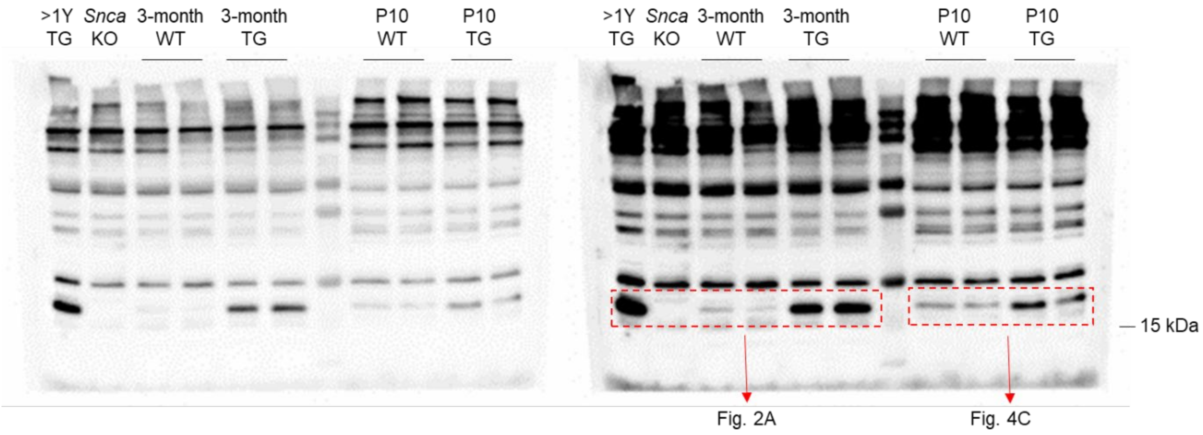

Western blot images for Fig. 2A and Fig. 4C. Full-length blot images with anti-p-α-Syn. Auto-exposure image is on the left and extended exposure image is on the right. The cropped images of each figure are marked with dotted red boxes.

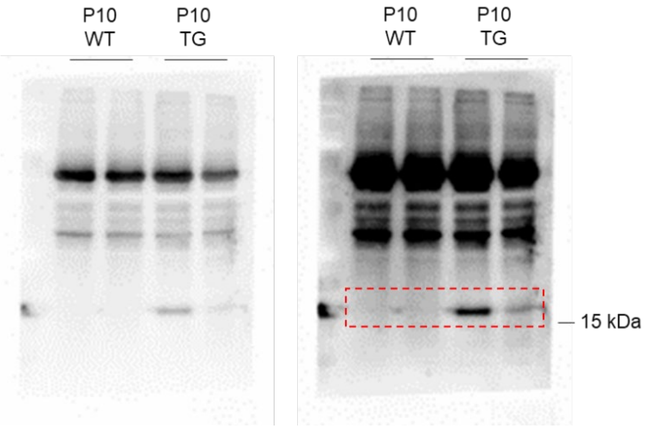

Western blot images for Fig. 4C. Full-length blot images with anti-α-Syn. Auto-exposure image is on the left and extended exposure image is on the right. The cropped image of the main figure is marked with dotted red boxes.

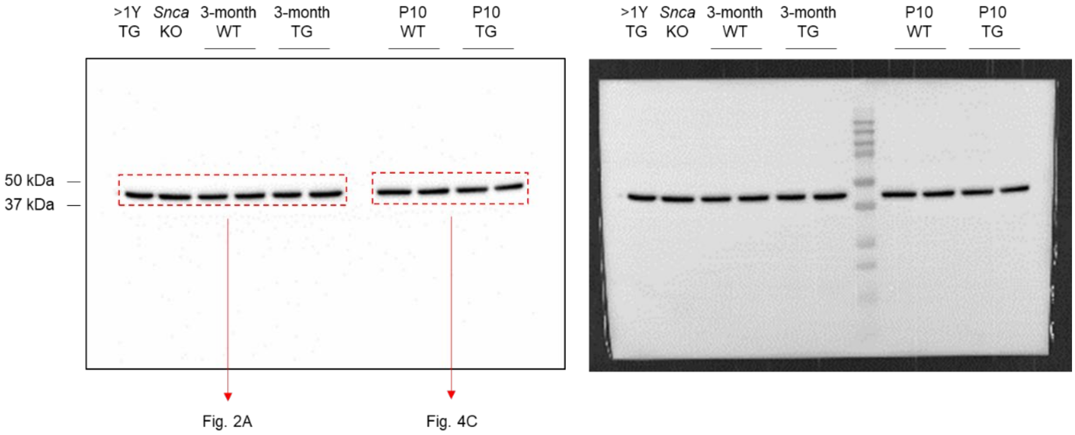

Western blot image for Fig. 2A and Fig. 4C. Full-length blot image with anti-β-actin before cropping on the left and merged image with protein size marker on the right side.

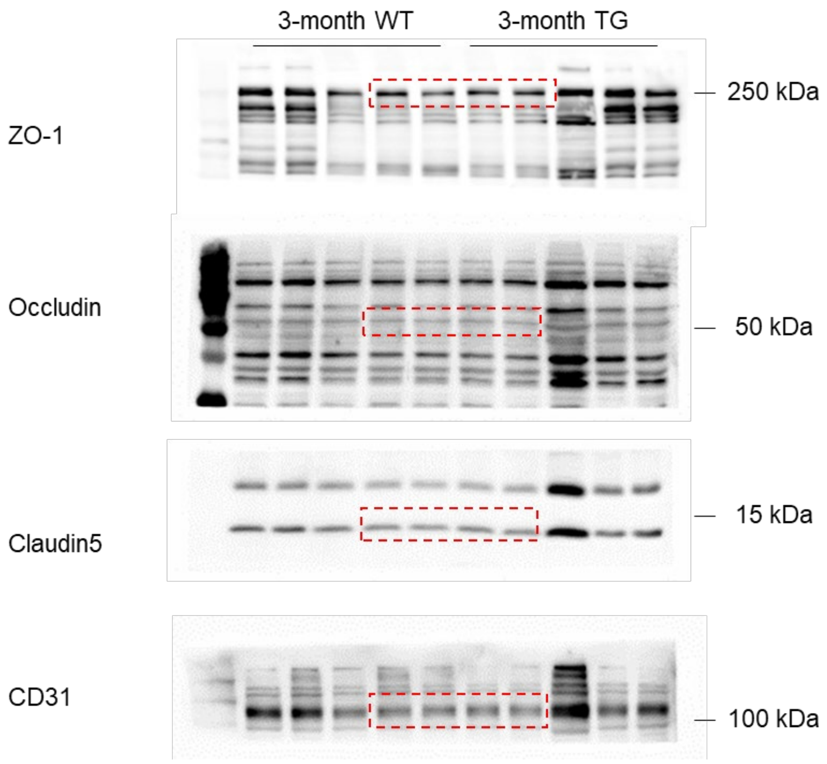

Western blot images for Supplementary Fig. 2C. Full-length blot images with anti-ZO-1, -Occludin, -Claudin5, and -CD31 from top to bottom. The cropped image of the main figure is marked with dotted red boxes. The sizes of each protein in the cropped images are compared with the information from the antibody companies.

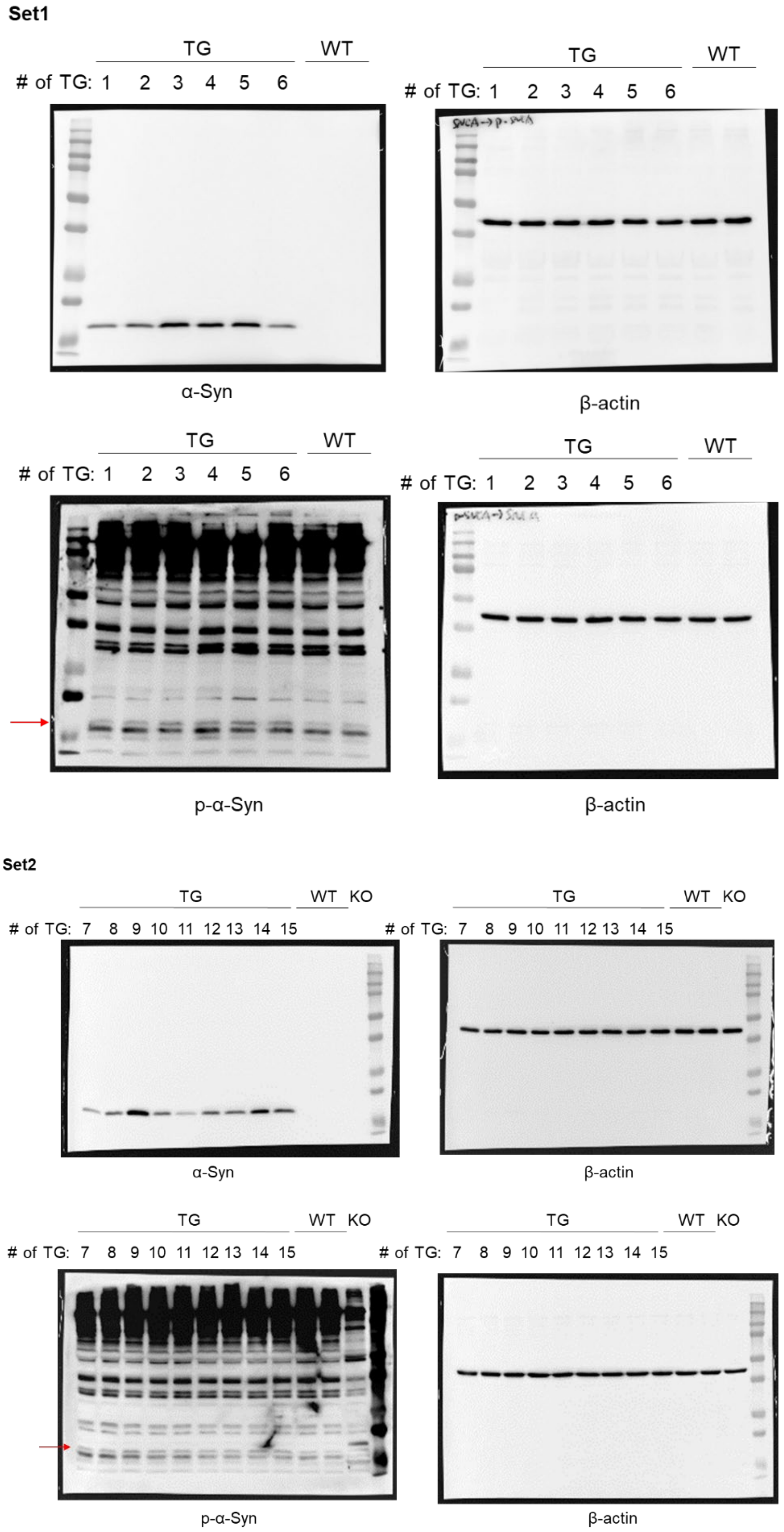

Western blot image for Supplementary Fig. 3. Full-length blot image with anti-α-Syn or anti-p-α-Syn and anti-β-actin probed after stripping. Both Set1 and Set2 were used for the quantification.

Full-length dot blot images

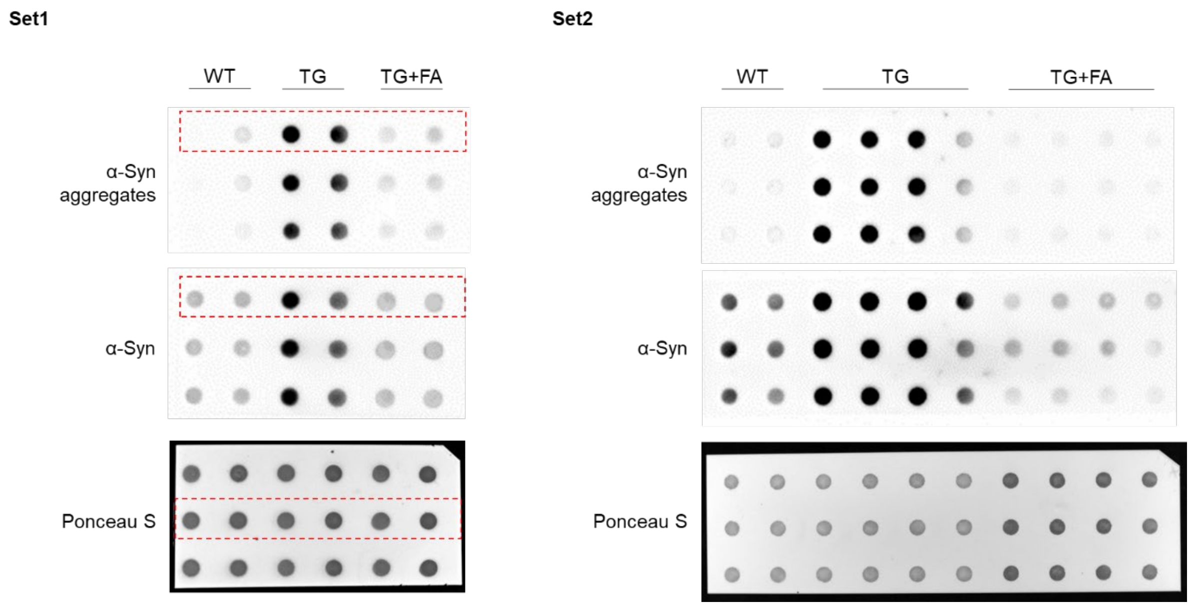

Dot blot images for Fig. 2B. Full-length dot blot images with α-Syn aggregate-specific or pan-α-Syn antibodies. The cropped image of the main figure is marked with dotted red boxes. Both Set1 and Set2 were used for the quantification.

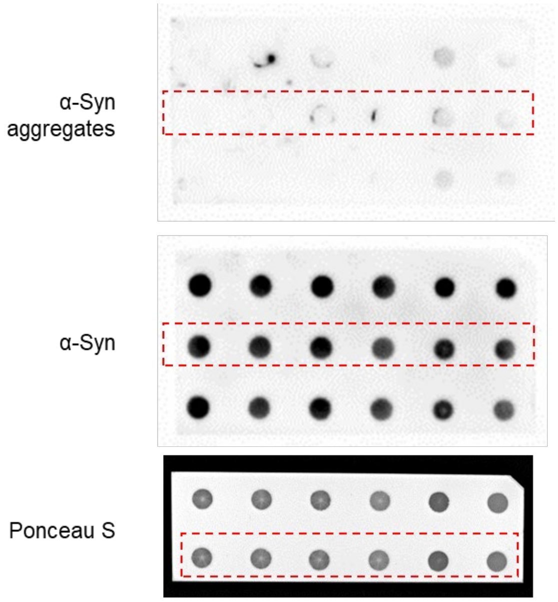

Dot blot images for Fig. 4D. Details are described in the above images.
